# Habitat suitability and distribution patterns of Rouget's rail (*Rougetius rougetii* Guérin‐méneville, 1843) in Ethiopia

**DOI:** 10.1002/ece3.70276

**Published:** 2024-09-12

**Authors:** Hailu Tilahun Argaw, Afework Bekele, Anagaw Atickem, Nils Chr. Stenseth, Diress Tsegaye, Bezawork Afework Bogale

**Affiliations:** ^1^ Department of Zoological Sciences, College of Natural and Computational Sciences Addis Ababa University Addis Ababa Ethiopia; ^2^ Department of Wildlife and Ecotourism Management, College of Agriculture and Natural Resource Wolkite University Wolkite Ethiopia; ^3^ Centre for Ecology and Evolutionary Synthesis University of Oslo Oslo Norway; ^4^ Department of Landscape Monitoring, Survey and Statistics Division Norwegian Institute of Bioeconomy Research Ås Norway

**Keywords:** climate change, ensemble models, Ethiopian highland, habitat suitability, Rouget's rail, threatened species

## Abstract

Geographical distribution and diversity patterns of bird species are influenced by climate change. The Rouget's rail (*Rougetius rougetii*) is a ground‐dwelling endemic bird species distributed in Ethiopia and Eritrea. It is a near‐threatened species menaced by habitat loss, one of the main causes of population declines for bird species. The increasing effects of climate change may further threaten the species’ survival. So far, the spatial distribution of this species is not fully documented. With this study, we develop current potential suitable habitat and predict the future habitat shift of *R*. *rougetii* based on environmental data such as bioclimatic variables, population density, vegetation cover, and elevation using 10 algorithms. We evaluated the importance of environmental factors in shaping the bird's distribution and how it shifts under climate change scenarios. We used 182 records of *R. rougetii* from Ethiopia and nine bioclimatic, population density, vegetation cover, and elevation variables to run the 10 model algorithms. Among 10 algorithms, eight were selected for ensembling models according to their predictive abilities. The current suitable habitats for *R. rougetii* were predicted to cover an area of about 82,000 km^2^ despite being highly fragmented. The model suggested that temperature seasonality (bio4), elevation, and mean daily air temperatures of the driest quarter (bio9) contributed the most to delimiting suitable areas for this species. *R. rougetii* is sensitive to climate change associated with elevation, which leads shrinking distribution of suitable areas. The projected spatial and temporal pattern of habitat loss of *R. rougetii* suggests the importance of climate change mitigation and implementing long‐term conservation and management strategies for this threatened endemic bird species.

## Abstract 2

*Rougetius rougetii* is a near‐threatened, ground‐dwelling endemic bird species distributed in Ethiopia and Eritrea, and the spatial distribution of this species is not fully documented. With the help of an ensembling model and the current distribution, which covers an area of ~82,000 km^2^, we develop potential suitable habitats now and forecast future habitat shifts based on environmental data. Long‐term conservation and management plans for this near‐threatened endemic bird species, as well as mitigating the effects of climate change, are crucial, as indicated by the anticipated spatial and temporal pattern of range shifts.

## 1 INTRODUCTION

Habitat suitability is important for the survival of organisms, reproduction, and population development (Matthiopoulos et al., 2015; Spear et al., 2010). Several environmental factors including local resource availability, elevation, and levels of habitat disturbance, determine the distribution pattern and degree of habitat suitability of different bird species (Dunning et al., 1992; Evans et al., 2009; Garden et al., 2006; Saab, 1999). Thus, understanding a species' biology and natural history should focus on studying the distribution in its habitat (Guisan et al., 2017). In this regard, species distribution models (SDMs) are used to predict how the world's biota will respond to climate change and other environmental factors. In addition, SDM helps to understand environmental predictors that could affect the range and distribution of species and also help identify where suitable habitats are distributed in space and time (Feng et al., 2019; Jetz et al., 2007). In the face of climate change, SDM maybe a critical tool for predicting future changes in the distribution of species and habitats and in the function of ecosystems.

Climate change is one of the main causes of change in the spatial arrangement of suitable habitats for bird species in either direct or indirect ways (Stewart et al., 2022). Limiting factors affecting species distribution have been studied by researchers on species dispersal ability (Dormann, 2007), land use type (Sirami et al., 2017), availability of resources, soil condition, topography, and landscape structure (Franklin, 2013). Birds may adapt to the effects of climate change by gradually shifting their geographic ranges to track better thermal conditions, even if the habitat type and other resources might not perfectly match their ecological requirements (Bosco et al., 2022). Birds have adapted to changing habitats through evolutionary processes (morphological or phenological adaptation) (Sexton et al., 2009). They can adapt morphologically by increasing or decreasing in body mass to regulate heat better, or phenologically by adjusting the time they perform life events like mating, reproduction, hibernation, and migration (McWhorter et al., 2018; Sauve et al., 2021).

Animals adapt when habitats and natural cycles alter due to climate change. It is this ability to adapt that allows the majority of species to evolve over millions of years. However, it has recently been suggested that contemporary plants and animals will not be able to adapt quickly enough to keep up with the rate of human‐driven climate change (Radchuk et al., 2019). This is because rapid climate change has the potential to affect survival or fitness by interfering with the energy required by organisms to maintain their basic levels of activity and conditions, reproduction, as well as their breeding and migratory timing (Hoffmann et al., 2019; Huey & Buckley, 2022; IPCC, 2021).

SDMs are used to understand how species' observed distribution is affected by a suite of environmental predictors and to subsequently predict how the potential distribution of the target species could change across space and time under different conditions, for instance, following climate change. SDMs consist of different tools and protocols that link the species presence to environmental factors like temperature and precipitation (Datta et al., 2020; Evans et al., 2009; Feng et al., 2019; Jetz et al., 2007; Thuiller et al., 2022). By studying the data and analyzing the distribution of a species, researchers can learn where the species may be located in the future in relation to climate change (Mason et al., 2019; Zuckerberg, 2017). It is known that global climate change is a rapidly occurring phenomenon (IPCC, 2021) and it is primarily driven by anthropogenic greenhouse gas emissions that change Earth's climate dynamics. This alteration has the potential to significantly impact organisms, from the genetic level to entire ecosystems. Specifically, climate change and intensive land use following population pressure are the major threats to high‐altitude species in both protected and unprotected areas (Israel et al., 2016).

To study such changes and estimate the conditions suitable for a species to inhabit a given area, statistical models have been used to connect known species locations to a suite of environmental variables. Different studies have found that SDMs can produce varied results based on different factors, such as the selected algorithms and predictors. It has been shown that no single SDM framework is best for all species and environments. Thus, it is important to be cautious when selecting the SDM to implement, because various modeling choices affect the accuracy of the obtained predictions. Comparison of multiple SDMs is fundamental to selecting the models, or the combination of models (i.e., ensemble modeling), permitting minimal prediction error (Guisan et al., 2017). Most studies compare more than one model to minimize error and get the best result (Brown et al., 2014; Zeng et al., 2016). Some studies advise using presence‐absence models, while others suggest presence‐only models. Despite these differences, the selection of suitable models depends on the type of application, the extent to which models are calibrated, and the spatial resolution of the environmental predictors.

The family Rallidae contains 152 species (Gill et al., 2020). Africa hosts six endemic genera of Rallidae, with 23 endemic species distributed all over the continent, except in the areas that are deserts and ice‐covered mountains. In Ethiopia, nine genera consisting of 12 extant species, some of which being resident, are reported; among them, Rouget's rail (*Rougetius rougetii* Guérin‐méneville, 1843) represents an endemic species of East Africa (Eritrea and Ethiopia) (Asefa et al., 2017). Rouget's rail population has drastically declined in the last few decades, and hence, it has been listed as near‐threatened by the International Union for Conservation of Nature (IUCN) since 1994 (http://www.birdlife.org). Currently, most Rallidae species inhabit a heavily degraded habitat, as they have lost much of their former suitable habitats and breeding sites (BirdLife International, 2023). In addition, they are observed in urban areas as feeding and breeding grounds along swampy and river streams with struggling anthropogenic pressures (BirdLife International, 2023).

We used SDMs for prioritizing conservation areas that can support a considerable population of this near‐endemic and threatened bird. In this regard, different individual distribution models were compared to predict the habitat suitability of *R. rougetii*. The present study gives empirical evidence on the current distribution of this species and the prediction of future distribution (2041–2070 and 2071–2100) in Ethiopia. Hence, the present study aims at (1) identifying the most important environmental variables influencing model predictions, (2) determining the potential distribution of *R. rougetii* in the current and future climate conditions, and (3) estimating the range change of *R. rougetii*, and synthesizing the potential effect of climate changes. To support the conservation efforts of this near‐threatened species, this study offers vital insights regarding the distribution and characteristics of *R. rougetii*'s natural habitats, highlighting both the species’ existing distribution and future appropriate habitats. Intending to efficiently reduce habitat loss and promote conservation efforts, this study forecasts future viable habitats in addition to mapping the current distribution.

## 2 MATERIALS AND METHODS

### 2.1 Occurrence data

**FIGURE 1:**
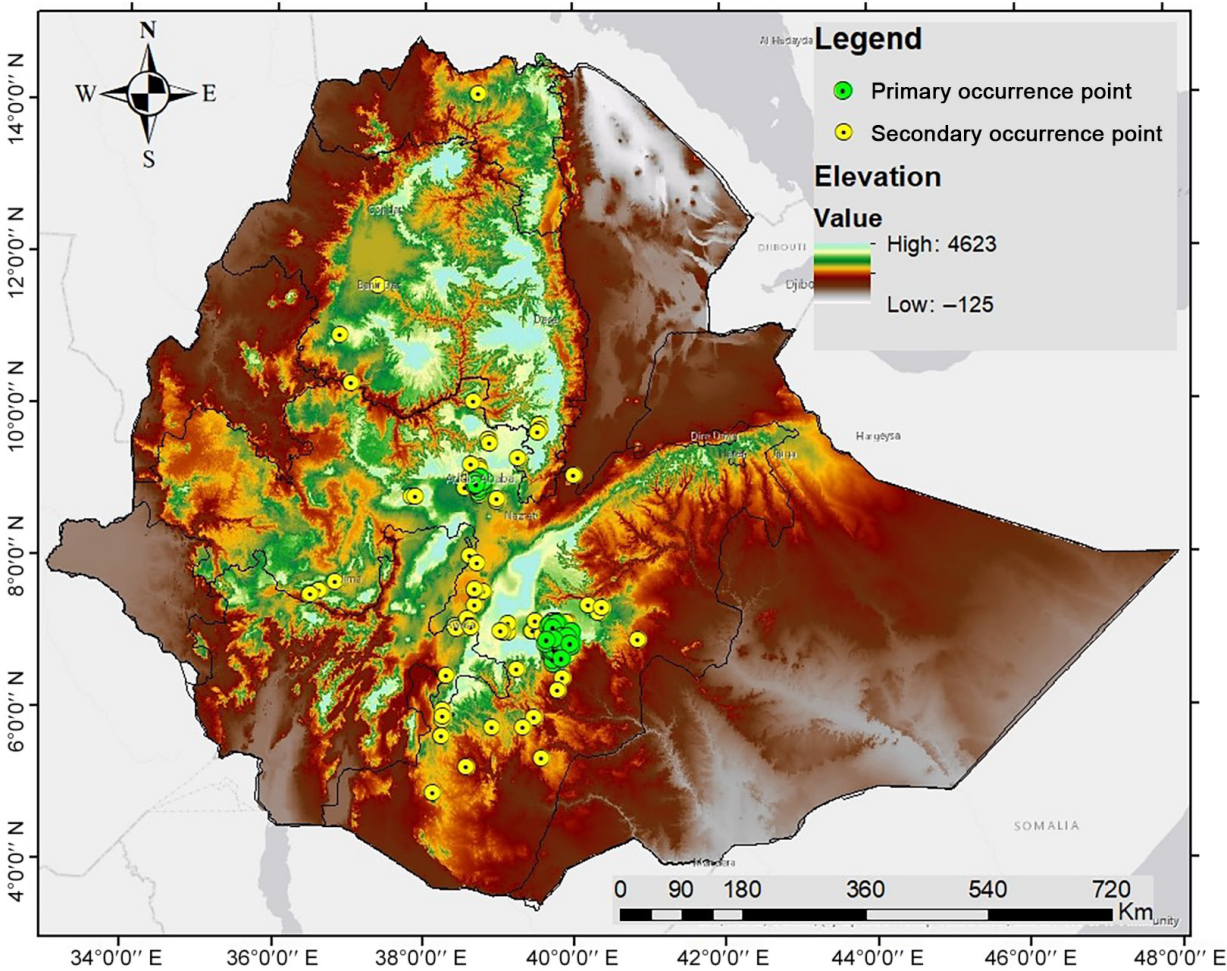
Maps showing *Rougetius rougetii* occurrences across elevation ranges of the study areas in Ethiopia.

We used primary and secondary occurrence data of *R. rougetii* to study the current habitat distribution and predict the future in Ethiopia using an ensemble modeling approach (Figure 1). The primary species occurrence data were recorded during field surveys (October, 2021–December, 2022) in the southeastern (6°29′–7°10’ N and 39°28′–39°58′ E) and central (8°45′–9°49′ N and 38°39′–38°54′ E) highlands of Ethiopia. A total of 98 occurrence points were recorded from the selected areas (Bale Mountains National Park, Goba town, and Addis Ababa city), and the minimum distance between the occurrence points was 1 km, which is aligned to a 1‐km^2^ raster grid resolution of bioclimatic variables.

Secondary occurrence data were downloaded from the online source Global Biodiversity Information Facility (GBIF) (http://www.gbif.org/). A total of 1161 occurrence points were downloaded and 84 occurrences were selected for this study. The GBIF data included occurrence records outside of the collection areas of primary data, and they were filtered by selecting only those recorded after 1981. We rarefied and aligned to a one‐kilometer square raster grid resolution of bioclimatic variables using “Spatially Rarefy Occurrence Data for SDMs” in the SDM Toolbox v. 2.5 (ArcGIS v. 10.7) to reduce spatial autocorrelation among all occurrence points. We randomly generated 10,000 pseudo‐absence points within the study area, excluding presence points using the Biomod2 package in R (Thuiller et al., 2022).

Overall, we generated a sample with data (SWD) by combining and rarefying 182 occurrences and 10,000 pseudo‐absence points to predict this bird species' current geographical distribution and future suitable areas. We used sufficient occurrence data because the predictive power of habitat suitability models is affected by numerous factors, of which occurrence data is one of the most important (Guisan et al., 2017).

### 2.2 Ecological predictor variables

We used 19 bioclimatic variables of current climate downloaded from Climatologies at high resolution for the earth's land surface areas (CHELSA) version 2.1 (available at http://envicloud.wsl.ch/) with the highest resolution of 30 arc seconds (~1 km), which are the average for the years 1981–2010 (Karger et al., 2017). Along with bioclimatic variables, we considered as candidate predictors the following factors: elevation, using the Shuttle Radar Topography Mission digital elevation model (SRTM‐DEM, https://srt.csi.cgiar.org/); human population, downloaded from https://www.ciesin.org/, as a proxy for human disturbance, which could be an influential variable for *R. rougetii* because the habitat used by this include urban areas; vegetation cover, downloaded from https://landscapeportal.org/layers/geonode:veg_ethiopia (see Table 1). To avoid the effect of multi‐collinearity among predictor variables, we used pairwise Pearson's correlation coefficients (*r* < |0.7|) (Guisan et al., 2017) and variance inflation factor (VIF < 10) (Thuiller et al., 2004). Accordingly, out of the total downloaded variables 12 variables were selected for this study (see summary in Table 1). Also, we downloaded the future climate projections under three shared socio‐economic pathway (SSP) scenarios (i.e. ssp126, ssp470, and ssp585), from CHELSA for two periods 2041–2070 and 2071–2100 at the same spatial scale resolution (30s arc‐seconds ~1 km^2^) of the current period data. The future model prediction only considers climate change constraints, using the same elevation, population and vegetation variables as the current suitable area prediction.

**TABLE 1:** Summary of selected ecological predictor variables used for modeling the habitat suitability of *Rougetius rougetii* with their category, code, unit of measurement, data sources and variance inflation factor (VIF). We included only those variables that show <10 VIF.

Category	Variables	Code	Units	Data sources	VIF
Bioclimatic	Mean annual air temperature range	bio2	°C	https://chelsa‐climate.org/bioclim/	1.213
	Isothermality	bio3	°C		1.277
	Temperature seasonality	bio4	°C/100		7.031
	Mean daily air temperatures of the driest quarter	bio9	°C		2.701
	Precipitation amount of the wettest month	bio13	kg m^−2^		5.540
	Precipitation amount of the driest month	bio14	kg m^−2^		3.932
	Precipitation seasonality	bio15	kg m^−2^		2.266
	Mean monthly precipitation amount of the warmest quarter	bio18	kg m^−2^		3.398
	Mean monthly precipitation amount of the coldest quarter	bio19	kg m^−2^		3.359
Population	Population	popu	Number	https://www.ciesin.org/	1.029
Vegetation	Vegetation cover	veg	Unitless	http://landscapeportal.org/layers/geonode:veg_ethiopia	1.170
Elevation	Elevation	elev	m	https://srtm.csi.cgiar.org/	7.282

### 2.3 Model fitting and evaluation

We used an ensemble modeling approach, to develop an accurate projection of suitable areas for *R. rougetii* in Ethiopia (Grenouillet et al., 2011; Marmion et al., 2009; Norberg et al., 2019; Oppel et al., 2012; Stohlgren et al., 2010). The presence and pseudo‐absence data were randomly split between a training set (70% of the data) used to calibrate the SDMs and a test set (the remaining 30%) used to evaluate model predictions. SDMs were built using 10 algorithms, including Generalized Linear Model (GLM), Generalized Boosting Method (GBM), Generalized Additive Model (GAM), Classification Tree Analysis (CTA), Artificial Neural Network (ANN), Surface Range Envelope (SRE), Flexible Discriminant Analysis (FDA), Multivariate Adaptive Regression Splines (MARS), Random Forests (RF), and Maximum Entropy (MAXENT) (Thuiller et al., 2022) (available at https://cran.r‐project.org/web/packages/biomod2.pdf). To evaluate the predictive performances of these algorithms, we used true skill statistics (TSS) and area under the receiver operating characteristics curve (AUC) (Allouche et al., 2006; Datta et al., 2020). Only the models showing TSS ≥0.7 and AUC ≥0.9 (Figure 3) were chosen to project habitat suitability for *R. rougetii* under present and future conditions, using the “BIOMOD_EnsebleModelling” function to build ensemble models (Marmion et al., 2009; Thuiller et al., 2004). We opted for two ensemble methods, namely committee averaging and weighted mean, in order to reduce the number of outputs and minimize the algorithm's bias effects in the individual predictions. However, upon comparing these ensemble options, it became evident that committee averaging provided a slightly better evaluation than the weighted mean option. Consequently, we chose committee averaging to present the results (Thuiller et al., 2022).

To estimate the change in the range distribution size of *R. rougetii*, the number of raster cells was counted and classified as the stables areas (suitable/unsuitable), loss and gain areas by comparing suitable habitats under current and future climate conditions. Ensemble predictions were binarized (i.e., suitable versus unsuitable cells), for both present and future projections, using a suitability value of 0.7 as threshold. Later, we used the BIOMOD_RangeSize function to count the number of stable, lost and gained suitable cells by comparing suitable habitats under current and future climate conditions (Datta et al., 2020; Guisan et al., 2017; Guo et al., 2021; Thuiller et al., 2022). All analyses were performed in R software version 4.2.2 (R Core Team) and followed standard protocol for reporting SDMs (Zurell et al., 2020).

## 3 RESULTS

### 3.1 Model performances and contributions of predictor variables

**FIGURE 2:**
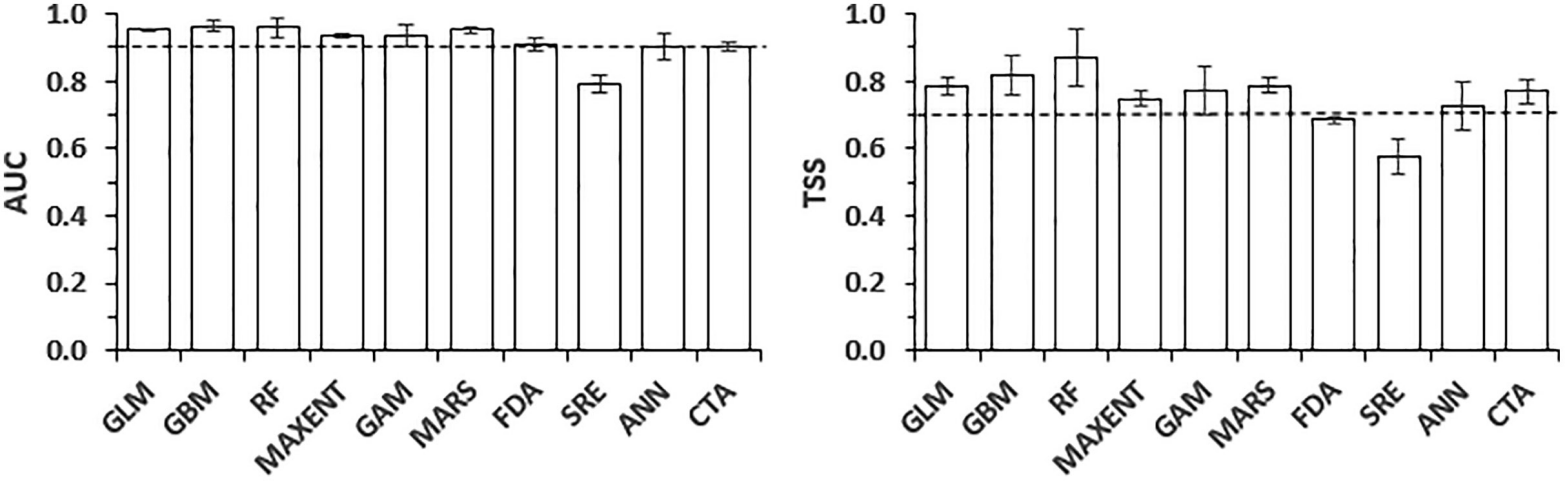
Evaluation of the predictive potential of 10 SDMs algorithms (*GLM*, *GBM*, *GAM*, *CTA*, *ANN*, *SRE*, *FDA*, *MARS*, *RF*, *MaxEnt*) for estimating habitat suitability of *Rougetius rougetii* based on True Skill Statistics (TSS) and Area under the receiver characteristic curve (AUC) scores.

The selected eight SDM algorithms (GLM, GBM, GAM, CTA, ANN, MARS, RF, MaxEnt) were used to build an ensemble model (evaluation metric quality threshold = 0.7) based on their good predictive performance using TSS and AUC values (Figure 2). The mean AUC varied between 0.79 ± 0.026 (*SRE*) and 0.96 ± 0.018 (*GBM*), and also the mean TSS varied between 0.58 ± 0.052 (*SRE*) and 0.87 ± 0.085 (*RF*). The *SRE* and *FDA* algorithms displayed weaker predictive performances than the others, excluding them from the final ensemble model (Figure 2).

**FIGURE 3:**
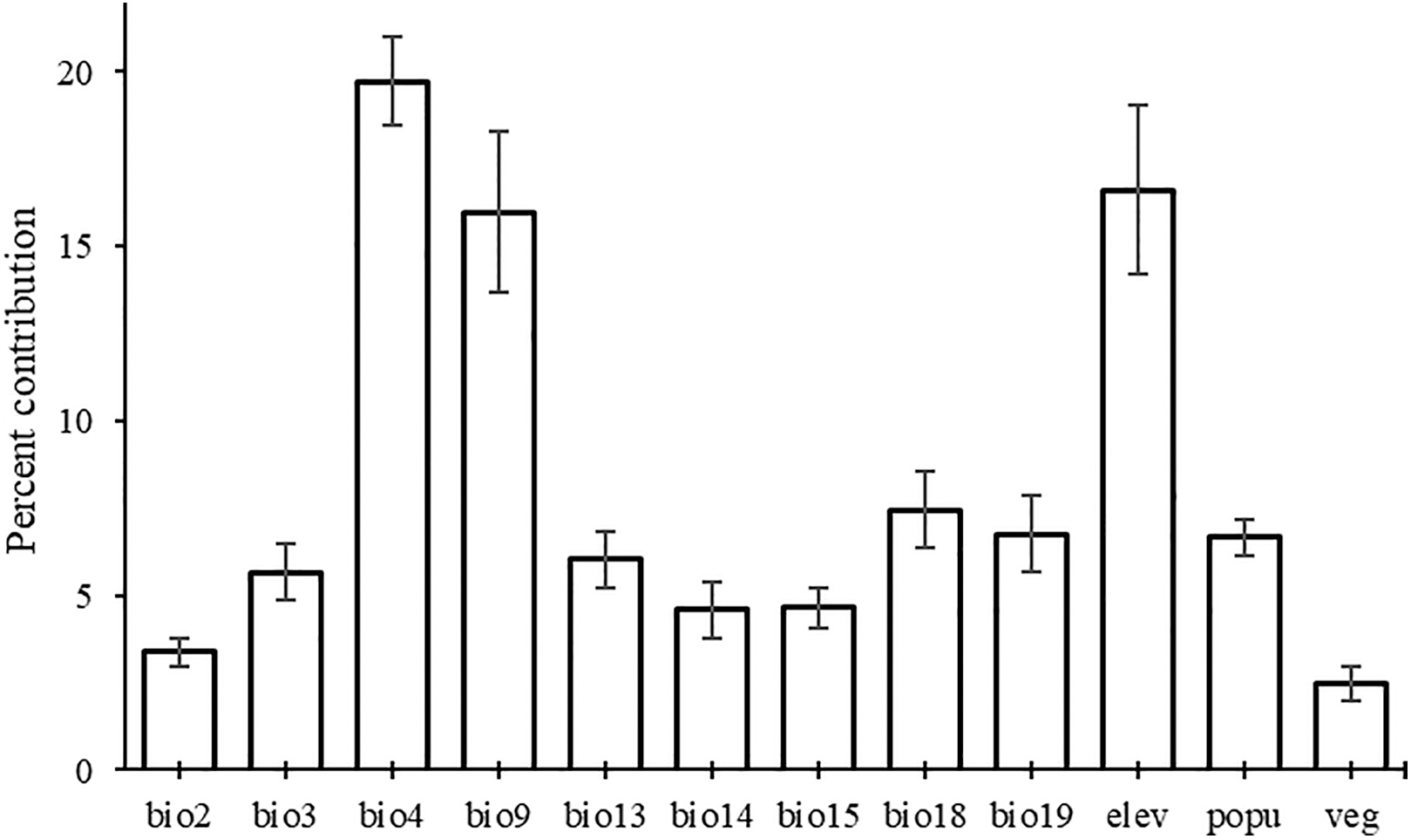
Percent contributions of the 12 selected predictor variables in the ensemble model of habitat suitability for *Rougetius rougetii*.

Temperature‐related factors' (total contribution of 44.75%) were the most influential in determining suitable conditions for *R. rougetii*, and the precipitation‐related factor contribution was the second, totaling 29.48%. Individually, the mean of variable importance by algorithm showed that bio4 (19.73 ± 1.28) was the most influential variable in limiting the habitat suitability of *R. rougetii*. The elevation (elev) and bio9 were the second and third most contributing variables in limiting the suitability of habitats of *R. rougetii* accounting for 16.62 ± 2.41 and 15.99 ± 2.30, respectively (Figure 3). Consequently, the elevation and temperature‐related factors were prominent factors in determining the habitat suitability of this threatened bird species.

### 3.2 Habitat suitability under present and future climates

**FIGURE 4:**
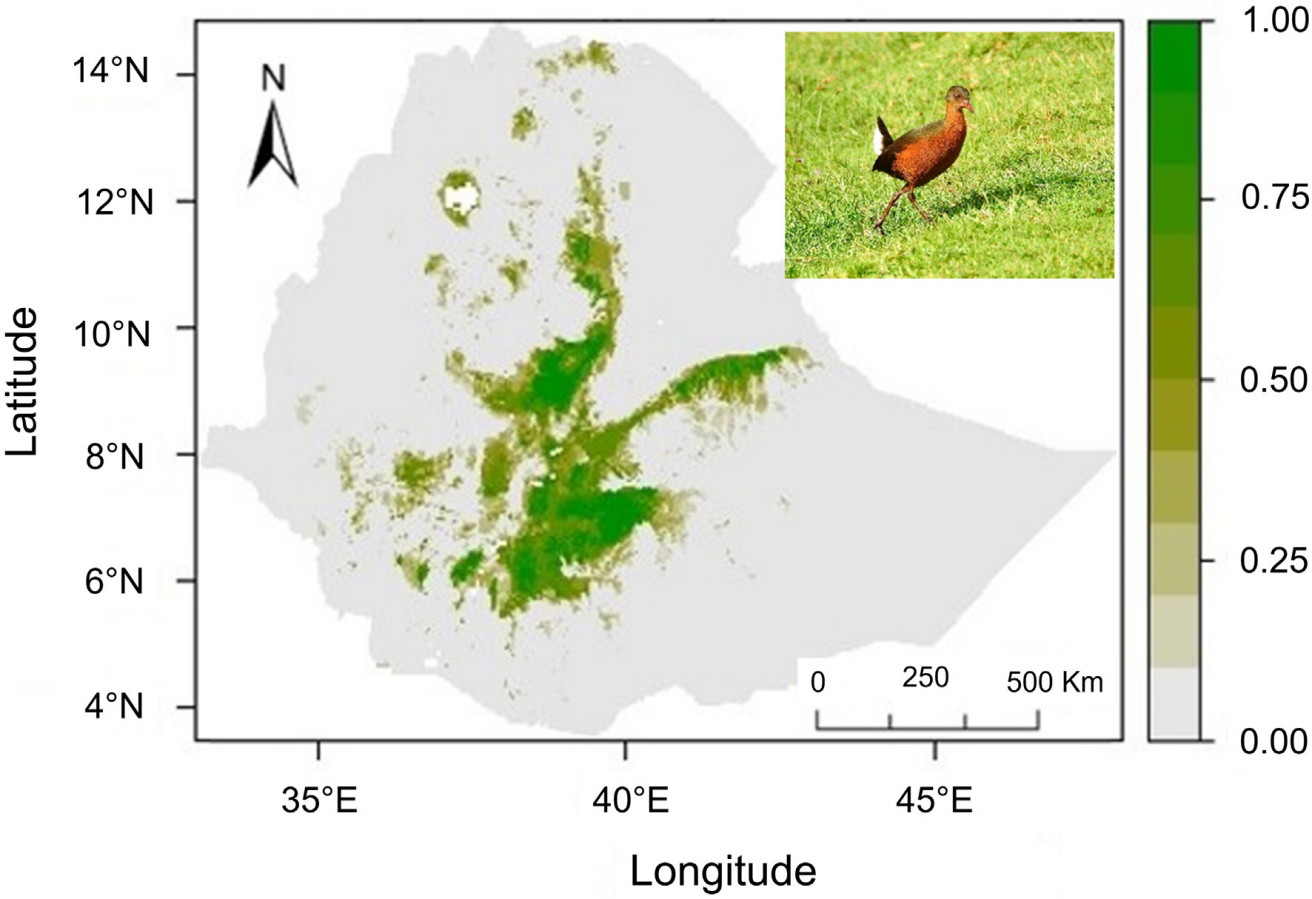
Predicted habitat suitability for *Rougetius rougetii* (shown in the inset picture) under current climate conditions.

According to the ensemble model projection, the currently suitable areas for *R. rougetii* are mainly located in the highlands of Ethiopia, covering almost 80,000 km^2^ (Figure 4). Several suitable areas were predicted in the eastern mountainous areas, most of the central highlands, and southeastern Ethiopia (the chain of Arsi‐Bale Mountains). Further, a continuous “core” suitable area emerged in most of the central and southeastern Ethiopian highlands. Some suitable habitats are shown to be fragmented in the southwest, the Central Rift Valley, and the northern region of the country.

**FIGURE 5:**
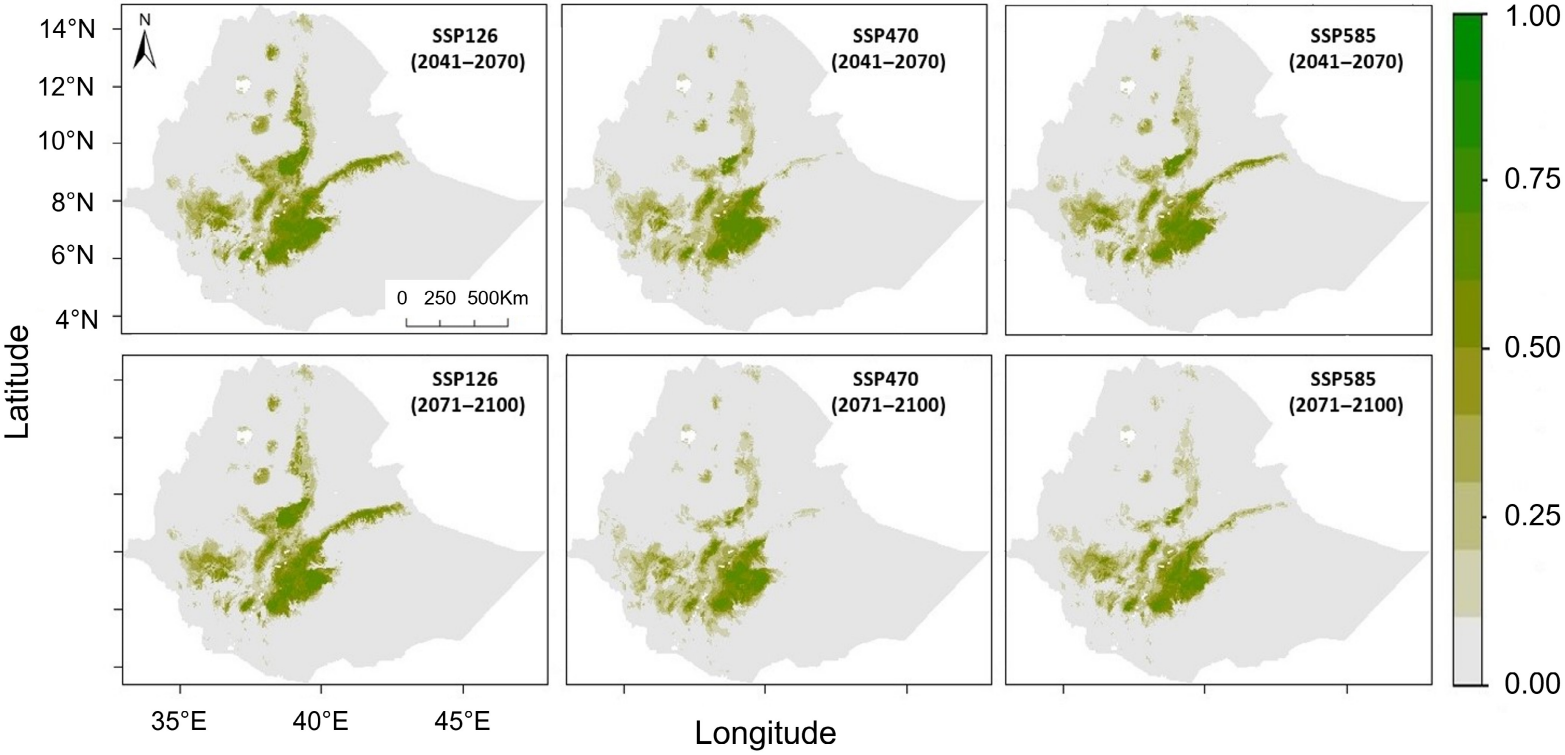
Predicted habitat suitability for *Rougetius rougetii* under future climate conditions in 2041–2070 and 2071–2100 was produced using SSP126, SSP370, and SSP585 scenarios.

The ensemble model projections suggested that the suitable areas for *R. rougetii* will be reduced significantly under both the future time horizons considered (2041–2070 and 2071–2100), with varying extent of lost suitable areas depending on the shared socio‐economic pathway scenarios (SSPs) used to predict future climatic conditions. The predicted suitable habitats of *R. rougetii* under SSP126, SSP370, and SSP585 covered about 61,000, 41,000, and 43,000 km^2^, respectively in 2041–2070 and 56,000, 35,000, and 36,000 km^2^, respectively in 2071–2100 (Figure 5; Table 2).

**TABLE 2:** Range size change in *Rougetius rougetii* under three shared socio‐economic pathway scenarios (SSPs) in 2056 and 2086.

Year	SSP	Area size (km^2^)			Area size (%)			
		Range size	Loss	Stable	Gain	Loss	Gain	Range change
1981–2010	Current	82915.26						
2041–2070	SSP126	60818.47	30298.60	52616.66	8201.81	36.54	9.89	−26.65
	SSP370	40692.59	46849.74	36065.51	4627.08	56.50	5.58	−50.92
	SSP585	43220.12	44019.46	38895.79	4324.33	53.09	5.23	−47.87
2071–2100	SSP126	55968.40	32846.71	50068.55	5899.85	39.62	7.12	−32.50
	SSP370	34761.87	51405.64	31509.62	3252.25	61.99	3.92	−58.07
	SSP585	34518.29	51108.89	31806.37	2711.92	61.64	3.27	−58.37

**FIGURE 6:**
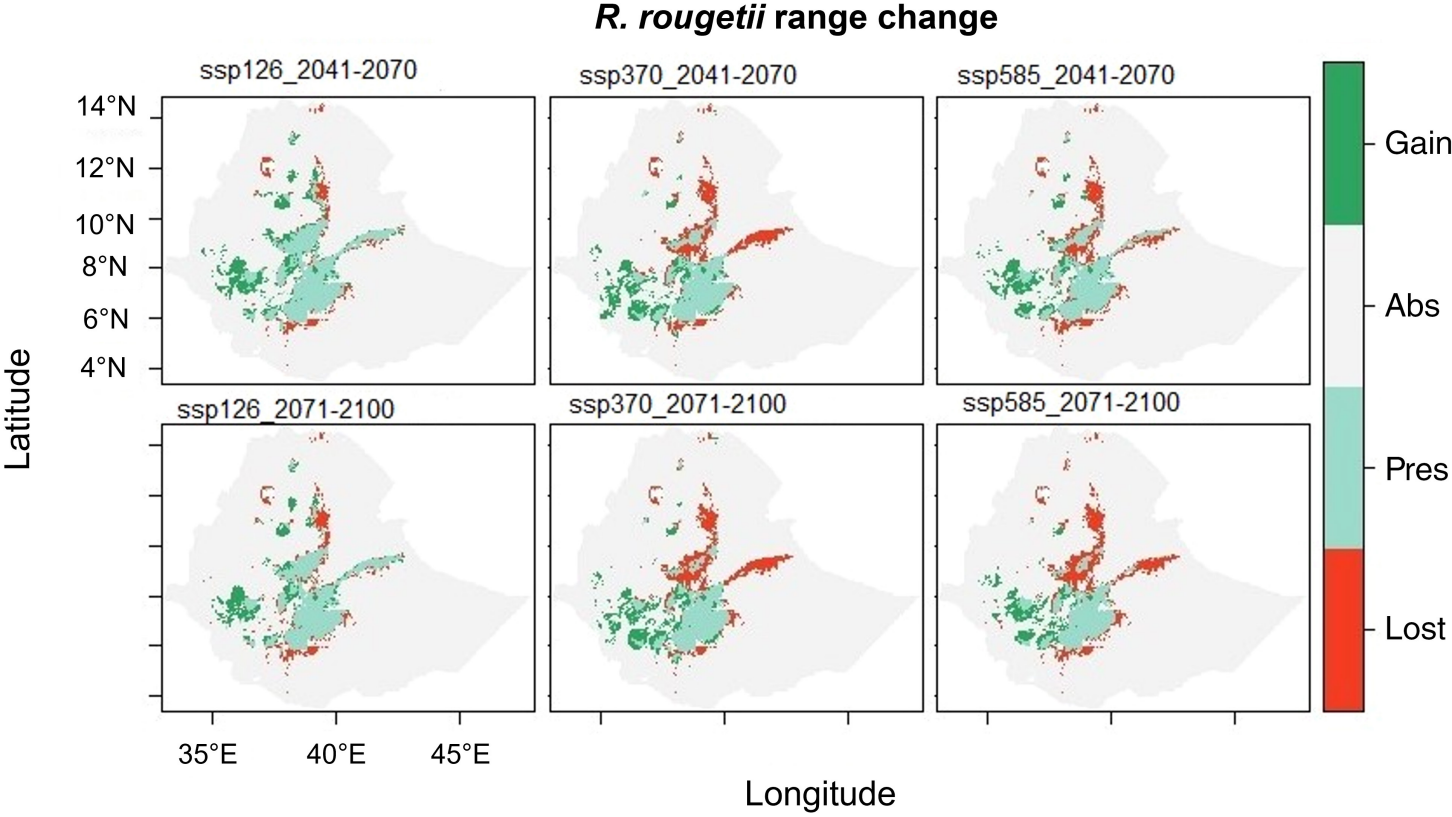
Current and future habitat suitability range changes in *Rougetius rougetii*; “lost” = habitat loss, “pres” = remain suitable, “abs” = remain unsuitable, and “gain” = unsuitable habitat changed to suitable.

The results of range size change in *R. rougetii* in 2041–2070 and 2071–2100 indicated a reduction under future climate conditions, and a big loss was observed in the northern part of the country and in lowland areas (Figure 6). Comparing current suitability maps with future suitability maps showed that significant portions of the suitable areas have been lost, reduced, and changed in the north, central, and eastern Ethiopian highlands.

The habitat suitability projections for *R. rougetii* under future climatic conditions varied based on the shared socio‐economic pathway scenarios (SSPs) used (Table 2). The highest loss in habitat range change is observed under SSP585 (58%) in 2071–2100 revealing a significant loss of suitable areas for this bird species from the current situation to future climate change.

## 4 DISCUSSION

This study examined the extent of suitable habitat of *Rougetius rougetii* under present and future climatic conditions. Furthermore, the variables influencing the geographical distribution of *R. rougetii* in Ethiopia were examined and ensemble models were built to predict current and future habitat suitability. A practical conservation strategy begins with discovering suitable habitats for vulnerable species (Zhang et al., 2012) such as *R. rougetii*. The ensemble model projection under current conditions showed that the suitable areas for *R. rougetii* in Ethiopia are far smaller than the geographic range reported for this species by BirdLife International (www.datazone.birdlife.org). On the basis of the current habitat suitability map, the species is likely to occur in Ethiopia's highlands and fragmented localities in the Central Rift Valley. The model predicted *R. rougetii* will lose a significant proportion of its suitable habitat in the future despite some newly suitable areas predicted to be gained. The results of this study demonstrate that *R. rougetii* is sensitive to climate change, since a significant portion of suitable habitat loss in its previous range was observed. The territorial bird species, particularly those with comparatively weak colonization potential, are poorly adapted to the changing climate and habitat fragmentation (Travis, 2003). This bird species may be in danger of extinction due to habitat loss under different climate scenarios (Ansley et al., 2023).

Among the 12 variables adopted in the model, bio4, bio9, and elevation have highly contributed to estimating the potential distribution of *R. rougetii*. This prediction is supported by a similar study in Great Britain where few climate change variables affected the distribution of bird species (Araújo et al., 2005). In the current study, in addition to climate change, other important variables such as elevation, and population also affected the potential distribution of the species. High human population density can affect the distribution of threatened bird species such as *R. rougetii*, which has a restricted range, poor ability to move, and a declining population, posing significant conservation challenges (Wuebbles et al., 2018).

Bird populations are declining globally and losing their suitable habitats due to climate change (Mason et al., 2019; Parmesan, 2006; Thackeray et al., 2010; Zuckerberg, 2017). Several SDMs have been developed to predict the suitability of their habitat under projected climatic conditions. Climate change is not only directly causing population declines for many bird species, but it may also promote new biotic interactions (Clavero et al., 2011). The high rate of increase in human population coupled with habitat disruptions may lead to the extinction of the species despite the presence of suitable habitats (CBD, 1992). Climate change interacts with and intensifies human modifications to the landscape, altering ecosystem structure and function, biodiversity, and species distributions (Rinawati et al., 2013).

Climate change may affect birds by changing the suitability of areas they currently inhabit forcing them to colonize new sites to find suitable habitats (Kuussaari et al., 2009). Finding such suitable habitats, however, depends on the dispersal ability of the bird species. Otherwise, birds may adjust phenologically or physiologically to cope with the challenge of climate change (Chmura et al., 2019; Quratulann et al., 2021; Scridel et al., 2018). If warming is neither too fast nor too pronounced, birds can adapt to rising temperatures through microevolution, i.e. by modifying the genetic structure of the population (Gienapp et al., 2008).

There is a significant difference between the suitable area for the Rouget's rail under the current and the future scenario. Compared to the current projection, more than half of the suitable area is lost in the future predictions, except in SSP126. It has been suggested that climate change may cause futureshifts in the distribution of biodiversity, with associated potential impacts on ecosystems (Bellard et al., 2012; Malhi et al., 2020). While climate change is predicted to cause range contraction for several species, it may also expand the suitable area for other species (Malhi et al., 2020; Martay et al., 2017). In the context of studies based on SDMs, the assumptions made regarding dispersion capacities of the target species and biotic interactions can have a substantial impact on the likelihood of model forecasts being verified in the future (Zuckerberg, 2017). We made the premise of limitless dispersion potential in our work, and our SDMs estimates suggest that the appropriate habitat for *R. rougetii* will probably diminish significantly, so that the new colonizable suitable areas in the future will not balance those being lost.

The present study suggests that *R. rougetii* is vulnerable to climate change, which will lead to a large range shift of suitable habitats under future climatic conditions. However, large amounts of currently suitable habitat may disappear because of land‐use change and habitat loss due to the rapid growth of the human population in the highlands of Ethiopia. Implementing long‐term conservation and management strategies in the *R. rougetii* distribution range, along with mitigation strategies to buffer climate change effects, is mandatory to preserve this threatened bird species from extinction.

## AUTHOR CONTRIBUTIONS

**Hailu Tilahun Argaw:** Conceptualization (lead); data curation (lead); formal analysis (lead); funding acquisition (lead); investigation (lead); methodology (lead); project administration (lead); resources (lead); software (lead); supervision (lead); validation (lead); visualization (lead); writing – original draft (lead); writing – review and editing (lead). **Afework Bekele:** Conceptualization (equal); project administration (lead); supervision (lead); validation (lead); writing – review and editing (equal). **Anagaw Atickem:** Formal analysis (equal); writing – review and editing (equal). **Nils Chr. Stenseth:** Writing – review and editing (supporting). **Diress Tsegaye:** Writing – review and editing (supporting). **Bezawork Afework Bogale:** Conceptualization (equal); project administration (equal); supervision (lead); validation (equal); writing – review and editing (equal).

## CONFLICT OF INTEREST STATEMENT

The authors declare that there is no conflict of interest.

## Data Availability

All important data are included in the main manuscript. The data supporting the findings is available upon request in the hands of the first (Hailu Tilahun Argaw) authors. Location records and environmental variables used to generate the models are made available on Dryad. https://doi.org/10.5061/dryad.547d7wmfq. Modeling procedures reported followed the ODMAP protocol (Zurell et al., 2020).
